# Atypical Subacute Cutaneous Lupus in a Patient on Apixaban Anticoagulation Therapy: A Case Report and Review of the Literature

**DOI:** 10.7759/cureus.16571

**Published:** 2021-07-22

**Authors:** Uros Rakita, Solomiya Grushchak, Lily Guo, Marylee Braniecki, Shilpa Mehta

**Affiliations:** 1 Dermatology, Chicago Medical School, Rosalind Franklin University of Medicine and Science, North Chicago, USA; 2 Department of Dermatology, John H. Stroger, Jr. Hospital of Cook County, Chicago, USA

**Keywords:** cutaneous lupus erythematosus, subacute cutaneous lupus erythematosus, apixaban, skin of color

## Abstract

Subacute cutaneous lupus erythematosus (SCLE) is a rare cutaneous lupus erythematosus (CLE) subtype manifesting in middle-aged Caucasians with photo-distributed papulosquamous or annular lesions. Drug-induced SCLE (DI-SCLE) forms present in a similar manner but direct oral anticoagulants are rarely implicated. We report an unusual case of SCLE in a 37-year-old African American patient with a history of unprovoked deep vein thromboses (DVT) who presented with new-onset photoprotected polymorphic lesions two months after the initiation of apixaban anticoagulation therapy. Our case demonstrates the heterogeneous nature of SCLE presentation and highlights the possibility of apixaban as a potential causative agent of DI-SCLE in immunogenetically susceptible individuals. Moreover, we hypothesize on the etiopathogenesis of our patient’s atypical presentation.

## Introduction

Subacute cutaneous lupus erythematosus (SCLE) is a rare cutaneous lupus erythematosus (CLE) subtype, and it usually manifests in middle-aged Caucasian individuals [1]. SCLE presents with photo-distributed papulosquamous or annular lesions and often fulfills systemic lupus erythematosus (SLE) diagnostic criteria, with limited systemic involvement [2]. We describe an unusual case of SCLE presenting in an African American female patient with photoprotected polymorphic lesions with onset two months after the initiation of apixaban anticoagulation therapy. Additionally, we review the related literature and hypothesize on the etiology and predisposing factors of her atypical presentation.

## Case presentation

**Figure 1:**
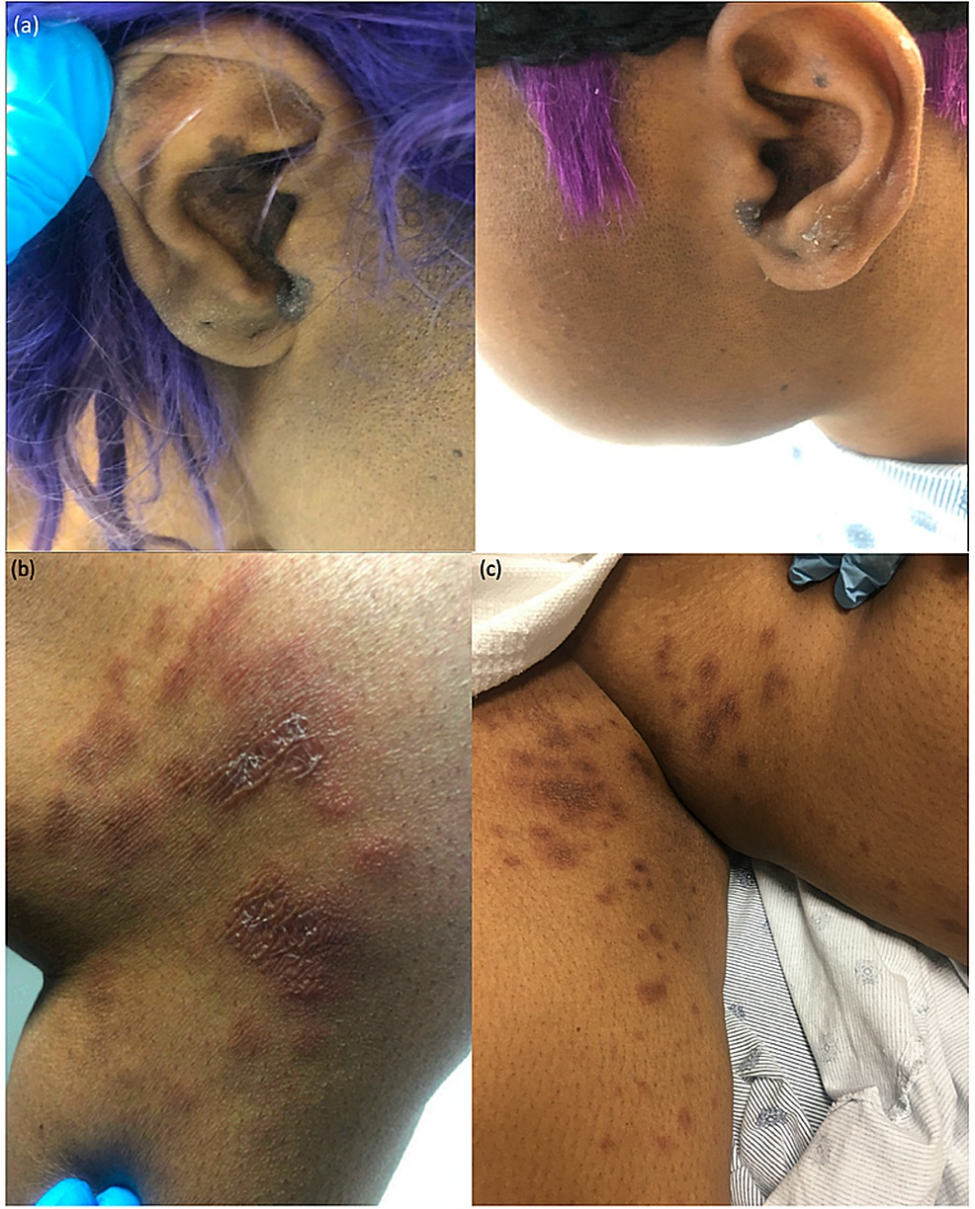
Clinical photographs (a) Hyperpigmented brown scaly plaques with central atrophic patches on bilateral conchal bowls. (b-c) Representative lesion of those found on the medial thigh, medial knee, and antecubital fossa. Red to violaceous nodules and plaques with scattered flaccid vesicles on (b) left and (c) right medial knees and thighs

A 37-year-old African American female with a history of two unprovoked deep venous thromboses (DVT) treated with apixaban was evaluated for a pruritic rash of two weeks' duration. She had started apixaban two months prior to the rash onset; there were otherwise no changes in her medication regimen. The exam showed hyperpigmented brown scaly plaques with central atrophic patches on the conchal bowl of the ears (Figure 1a), violaceous nodules, and indurated plaques with scattered flaccid vesicles on the medial thighs, knees, and antecubital fossae (Figures 1b, 1c), and eroded plaques with dry, serosanguinous crust on the elbows. Vesicles were not appreciated on non-lesional skin and there was no mucosal involvement. She endorsed hematuria and weight loss but denied lesion photosensitivity. Laboratory analysis was significant for leukopenia (WBC of 3.4 thousands/μL), anemia (hemoglobin of 5.6 g/dL), and proteinuria. Serology was positive for antinuclear antibodies (ANA) (>1:160), anti-double-stranded DNA (anti-dsDNA), anti-Smith, anti-Ro/SSA, cardiolipin antibody, beta2 glycoprotein antibody, and low C3 and C4 complement.

**Figure 2:**
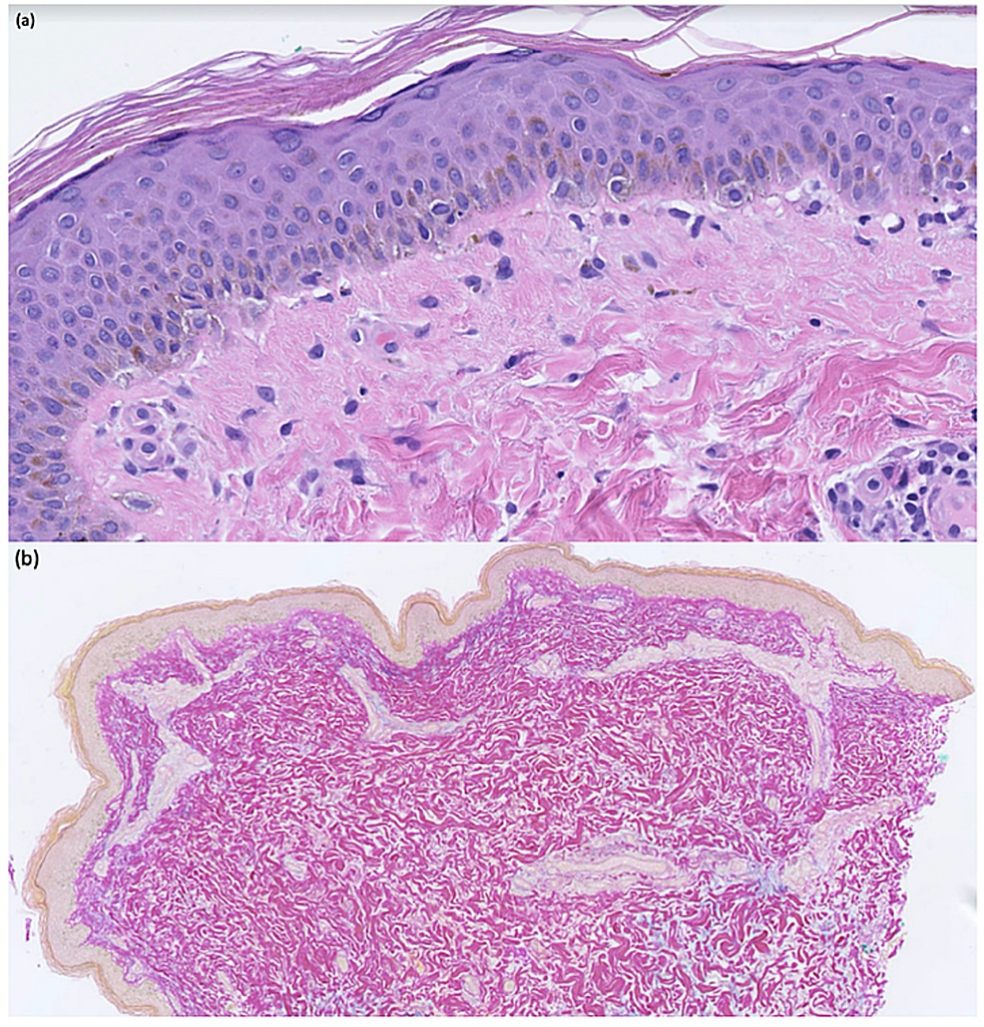
Skin histopathology (a) Early vacuolar degeneration of basal epidermal layer and scant dermal perivascular lymphocytic infiltrate (hematoxylin and eosin stain, original magnification x40). (b) Mucin deposition in superficial and deep dermal layers (colloidal iron stain, original magnification x10)

A 4-mm punch biopsy of a representative lesion from the medial knee was performed. Histopathologic findings suggested early SCLE and consisted of basilar vacuolar degeneration with scant dermal perivascular lymphocytic infiltrate (Figure 2a). Colloidal iron staining highlighted mucin deposition throughout the superficial and deep dermis (Figure 2b). Apixaban was discontinued and warfarin initiated. Triamcinolone 0.01% was used as needed for her skin. She remains in remission at her three-month follow-up, treated with hydroxychloroquine (200 mg BID), mycophenolate mofetil (1 gm BID), and prednisone (40 mg daily) for her SLE and lupus nephritis, respectively.

## Discussion

CLE comprises three subtypes for which no standardized classification criteria exist [2]. The SCLE subtype occurs most commonly in middle-aged Caucasian females, and those presenting with SCLE roughly represent 10-15% of lupus patients in a given locale [1]. Typical lesions are papulosquamous or scaly, annular photodistributed plaques that paradoxically spare the midface while involving the lateral face, trunk, and extensor aspects of the upper extremities [1,3]. Rare, vesiculobullous SCLE variants primarily present with erosions and crusting with occasional vesicles at the active periphery or central plaque areas [4]. Compared to lesions of the more common discoid lupus erythematosus (DLE), SCLE lesions are more photosensitive [1]. Also, unlike DLE lesions, SCLE ones are nonscarring and nonindurated, representing a more superficial pathology with a relatively sparse inflammatory infiltrate [1,2]. Of note, patients can concurrently present with multiple CLE subtypes. Roughly 20% of SCLE patients develop DLE lesions during their disease course [1]. Moreover, up to half of SCLE patients also meet the criteria for SLE; however, serious systemic involvement is infrequent [2].

There is significant histologic overlap between CLE phenotypes, and their differentiation on the basis of histology alone is unreliable. Common CLE findings include interface dermatitis with mononuclear cell infiltrate at the dermoepidermal junction, basal cell degeneration, perivascular and periadnexal inflammation, dermal mucin deposition, and hyperkeratosis [3]. A superficial infiltrate with a milder histological presentation of the above features is suggestive of SCLE [2]. Serologic associations add diagnostic clarity since, unlike other CLE variants, SCLE is highly associated with anti-Ro/SSA antibodies with 70% of patients testing positive [2]. On the other hand, the SLE-specific antibodies (anti-dsDNA and anti-Smith) are relatively rare in SCLE patients [1,2].

As SCLE is rare in African Americans [1], our patient’s ethnicity, the indurated and vesicular presentation of her rash, as well as the presence of lesions in photoprotected areas were reasons this case was atypical and diagnostically challenging. Interestingly, anti-Ro/SSA antibodies may serve a photoprotective role in patients with skin of color (SOC) [5]; however, this may only be limited to specific ethnicities in SOC patients [6]. This suggests that certain ethnic groups with SCLE may have an atypical presentation. Areas of vesiculation perhaps represented accentuations of interface dermatitis [4]. Vesicular lupus lesions lack uniform classification [4]; however, given our patient’s histologic and serologic findings, the etiology is likely related to the underlying lupus pathology. Although not histologically examined, the co-occurrence of characteristic DLE lesions in the conchal bowl raised our suspicion for lupus erythematosus. Antibody profiles had features specific for both SCLE and SLE. This in conjunction with renal, hematologic, and immunologic involvement indicate that our patient belongs to the minority of SCLE patients meeting SLE diagnostic criteria with notable systemic disease.

Moreover, it is possible that her new-onset SCLE was related to the recently initiated apixaban therapy. Drug-induced SCLE (DI-SCLE) is clinically as well as serologically indistinguishable from the idiopathic form and can occur days or years after drug initiation [7]. Unlike drug-induced SLE, DI-SCLE is not typically associated with anti-histone antibodies [8].

A wide range of drugs have been associated with DI-SCLE; however, direct oral anticoagulants are rarely implicated. McCarthy et al. have published a case of rivaroxaban-induced SCLE, which presented with typical clinicopathologic features three weeks after the drug administration and resolved within four months of drug discontinuation and concurrent topical corticosteroid therapy [9]. Recently, apixaban was implicated in anti-histone antibody-positive drug-induced SLE with cutaneous manifestation consistent with leukocytoclastic vasculitis [10]. Rash appearance in the aforementioned case occurred 15 days after the drug initiation. Considering the temporal association between SCLE appearance and apixaban administration along with improvement upon apixaban discontinuation, we believe apixaban therapy was possibly associated with SCLE precipitation in our patient. Given our patient’s DVT history, we hypothesize that apixaban may have exacerbated her baseline immunogenetic susceptibility to SCLE.

## Conclusions

We presented a patient with SCLE with newly diagnosed SLE. Our patient displayed features spanning the lupus erythematous spectrum, which along with atypical findings makes this case unique and equally informative. It is important to emphasize that the hypothesized relationship between our patient’s ethnicity and photoprotected SCLE distribution, as well as the possible association between apixaban and DI-SCLE, are both merely speculative and require further investigation.
